# CLARITY reveals dynamics of ovarian follicular architecture and vasculature in three-dimensions

**DOI:** 10.1038/srep44810

**Published:** 2017-03-23

**Authors:** Yi Feng, Peng Cui, Xiaowei Lu, Brian Hsueh, Fredrik Möller Billig, Livia Zarnescu Yanez, Raju Tomer, Derek Boerboom, Peter Carmeliet, Karl Deisseroth, Aaron J. W. Hsueh

**Affiliations:** 1 Departments of Obstetrics and Gynecology, Stanford University School of Medicine, Stanford University, Stanford, CA 94305, USA; 2 Department of Integrative Medicine and Neurobiology, State Key Lab of Medical Neurobiology, School of Basic Medical Sciences, Shanghai Medical College, and Institute of Brain Science, Brain Science Collaborative Innovation Center, Fudan University, Shanghai 200032, China; 3 Department of Bioengineering, Howard Hughes Medical Institute, CNC Program, Department of Psychiatry and Behavioral Sciences, Stanford University, Stanford, CA 94305, USA; 4 Department of Bioengineering, Stanford University, Stanford, CA 94305, USA; 5 Centre de Recherche en Reproduction Animale, Université de Montréal, St-Hyacinthe, Québec, Canada; 6 Laboratory of Angioenesis and Vascular Metabolism, Vesalius Research Center, VIB and Department of Oncology, University of Leuven, Leuven, Belgium

## Abstract

Optimal distribution of heterogeneous organelles and cell types within an organ is essential for physiological processes. Unique for the ovary, hormonally regulated folliculogenesis, ovulation, luteal formation/regression and associated vasculature changes lead to tissue remodeling during each reproductive cycle. Using the CLARITY approach and marker immunostaining, we identified individual follicles and corpora lutea in intact ovaries. Monitoring lifetime changes in follicle populations showed age-dependent decreases in total follicles and percentages of advanced follicles. Follicle development from primordial to preovulatory stage was characterized by 3 × 10^5^-fold increases in volume, decreases in roundness, and decreased clustering of same stage follicles. Construction of follicle-vasculature relationship maps indicated age- and gonadotropin-dependent increases in vasculature and branching surrounding follicles. Heterozygous mutant mice with deletion of hypoxia-response element in the vascular endothelial growth factor A (VEGFA) promoter showed defective ovarian vasculature and decreased ovulatory responses. Unilateral intrabursal injection of axitinib, an inhibitor of VEGF receptors, retarded neo-angiogenesis that was associated with defective ovulation in treated ovaries. Our approach uncovers unique features of ovarian architecture and essential roles of vasculature in organizing follicles to allow future studies on normal and diseased human ovaries. Similar approaches could also reveal roles of neo-angiogenesis during embryonic development and tumorigenesis.

Follicles are basic functional units of the ovary. Although most follicles remain at the primordial stage, activation of these dormant follicles leads to their growth into primary, secondary, and antral stages^1^. Among ~20 early antral follicles present during early follicular phase of the menstrual cycle in women, most become atretic and only one reaches antral/preovulatory stages. This dominant follicle secrets a majority of circulating sex steroids necessary for maintaining reproductive cyclicity and eventually ovulates a mature oocyte capable of developing into a new life^2^,^3^. After rupture, ovulated follicles become corpora lutea that secret progesterone important for pregnancy maintenance. Follicle growth, massive atresia, and rupture as well as luteal formation/regression are hormonally regulated and associated with neo-angiogenesis that is rarely found in adult mammals^4^. Thus, cyclic remodeling of ovarian structures takes place during each reproductive cycle accompanied by a lifelong decline of follicles until reproductive senescence^5^.

Many organs in vertebrates consist of compartmentalized branching units such as lobules in lung, liver, and mammary gland or tubules in testis and kidney. However, ovaries contain primordial follicles situated in the cortical region together with growing follicles arranged in a centripetal fashion without a clear pattern of organization^6^. Most traditional histological analyses deal with limited information regarding interrelationships among follicles and corpora lutea due to uncharacterized three-dimensional (3D) architecture. Based on nuclear volume of the oocyte as an indicator of follicles, a recent whole mount immunofluorescence 3D imaging study begun to provide follicle dynamics from neonate to adult mice^7^. However, there is no analysis on follicular somatic cells together with oocytes in 3D architecture. The vascular system is generally quiescent in adult mammals and the ovary and endometrium are the only normal adult tissues capable of undergoing neo-angiogenesis^8^. Furthermore, the relationship between folliculogenesis and ovarian vasculature is unclear.

Recently developed CLARITY approach makes intact tissues transparent and enables immunostaining of markers to elucidate detailed 3D structure of organs^9^. Using specific markers and advanced computer algorithms, we imaged ovarian follicles and corpora lutea in intact ovaries and generated 3D digital maps of ovarian organelles in relation to vasculatures to reveal changes in follicle sizes, location, roundness, clustering, interrelationships, and vasculature throughout reproductive life. In mutant mice with deletion of the hypoxia-response element in the vascular endothelial growth factor A (VEGFA) promoter, defective ovarian vasculature was found to be associated with lower ovulatory responses following gonadotropin stimulation. Following treatment with an inhibitor for VEGF receptor kinases, we also suppressed gonadotropin-induced ovarian neo-angiogenesis, leading to defective vasculature and ovulation. The purpose of the current study was to use the CLARITY approach to elucidate folliculogenesis and the relationship between vasculature and follicles in mice after birth to adult life.

## Results

### Identification of follicles and corpora lutea at different developmental stages

We used the CLARITY approach to render ovaries transparent (Figure S1A), followed by staining with different markers before digital imaging (Figure S1B). Our preliminary data (Figure S2) indicated that staining with antibodies against tyrosine hydroxylase (TH), the rate-limiting enzyme in catecholamine biosynthesis, led to prominent staining in theca cells, ganglia, and corpora lutea as confirmed using antibodies against neuron-specific class III beta-tubulin (Tuj1)^10^ and brain-derived neurotropic factor (BDNF), consistent with earlier findings^11^,^12^. In addition, nonspecific background TH staining was found around all follicles, allowing for identification of follicular structures. As shown in Figure S1B, primordial and primary follicles both showed TH staining but could be separated based on shapes of granulosa cells (squamous for primordial; cuboidal for primary). In secondary follicles, anti-Müllerian hormone (AMH) staining was evident in granulosa cells as reported^13^. Also, BID (a pro-apoptosis protein) and CYP19 (the enzyme for estrogen biosynthesis) marked granulosa cells in atretic^14^ and antral/preovulatory follicles^15^, respectively (Figure S1B). Preovulatory and antral follicles were detected semi-manually according to their sizes and the antral shape and location. In addition to their unique homogenous texture, corpora lutea showed staining for VEGF important for angiogenesis^8^. Also, digital slicing of adjacent images showed an ovulating follicle (Figure S1B).

### Three-dimensional determination of follicle numbers throughout life

**Figure 1:**
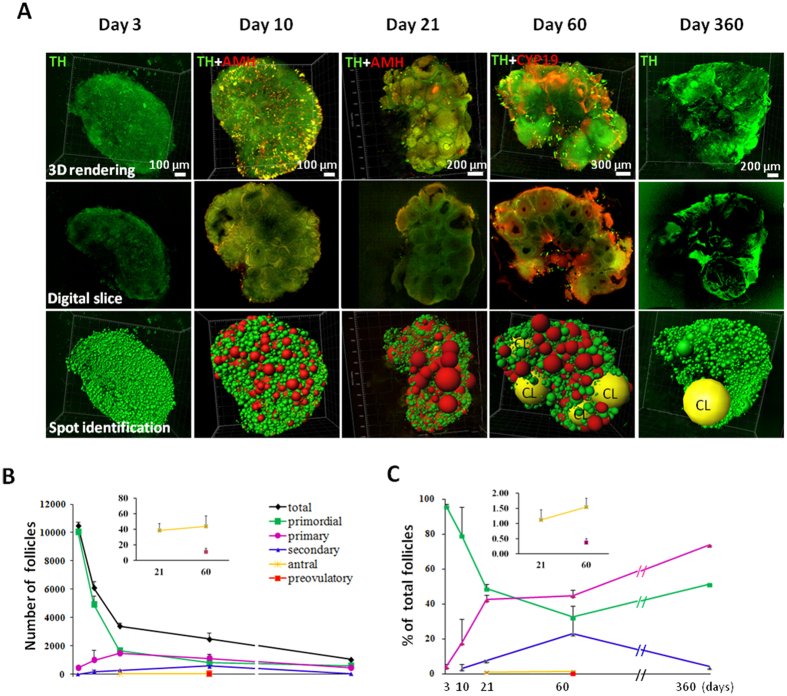
Identification of follicles at different stages and determination of follicle numbers throughout life . (**A**) Ovaries from mice at different ages were obtained for CLARITY processing and immunostaining using marker antibodies, followed by data transformation using the Imaris Spot algorithm. Upper panel: 3D rendering of whole ovary images; Middle panel: Digital slices showing follicles at individual planes; Lower panel: Data transformation into Spot graphs following identification of follicles using specific markers. Background staining using tyrosine hydroxylase (TH) antibodies provided outlines of all follicles whereas staining using antibodies to anti-Müllerian hormone (AMH) and aromatase (CYP19) allowed identification of follicles at secondary and antral/preovulatory stages. CL, corpus luteum. (**B**) Age-dependent changes in number of follicles at different developmental stages. (**C**) Age-dependent changes in percentages of follicles at different developmental stages. Means + SE are shown.

We analyzed follicle dynamics of intact ovaries from mice at different ages following CLARITY processing and immunostaining for TH together with AMH or CYP19 staining (Figure 1). Scanned images were analyzed following 3D rendering, digital slicing, and Spot transformation to identify primordial, primary, secondary, antral, and preovulatory follicles (Fig. 1A and Figure S3). In addition, corpora lutea were distinguished based on their unique homogenous texture in ovaries of adult and aging (day 360) mice. Three-D images of a day-10 mouse ovary were generated for Spot transformation, followed by manual improvement, to identify all follicles (Movie S1). Using specific markers, follicles at all developmental stages were traced together with corpora lutea in the ovary of an adult proestrous animal to construct follicle relationship maps (Movie S2).

Based on specific markers, we estimated number of follicles at different stages of development (Fig. 1) and determined their diameters, surface areas, and volumes (Table S1). Follicle volume increased ~45 fold during transition from primordial to primary stage whereas secondary follicle development encompassed 125-fold increases in volume, indicating major growth (Tables S1 and S2). From early antral to the largest preovulatory follicles, ~15-fold increases in volume were found. Throughout folliculogenesis from the smallest primordial to largest preovulatory follicles, ~3 × 10^5^-fold increases in follicle volume were found, accompanied by 88,000-fold increases in oocyte volume. Analyzing lifetime changes in follicle numbers indicated an age-dependent decline in total follicles, together with major decreases in primordial follicles (Fig. 1B) as previously reported^16^. Of interest, percentages of both primordial and primary follicles increased with age (Fig. 1C) accompanied by a drop of secondary follicles, suggesting age-dependent follicle arrest before the secondary stage.

### Clustering and migration of follicles based on 3D mapping

**Figure 2:**
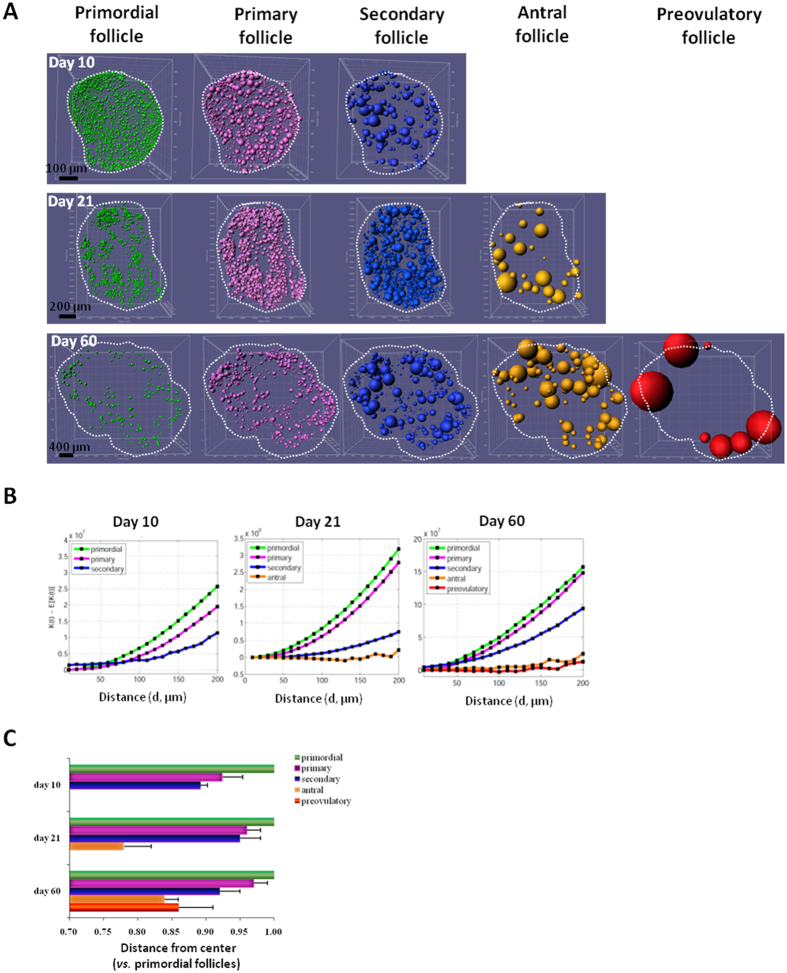
Construction of 3D follicle maps indicates follicle clustering and migration. (**A**) Spot representation shows location of follicles in ovaries from mice at 10, 21, and 60 days of age. Follicles at primordial (green), primary (pink), secondary (purple), antral (orange), and preovulatory (red) stages are shown. Note absence of antral and preovulatory follicles in day 10 ovary whereas day 21 ovary lacks preovulatory follicles. This graph has been drawn by Imaris Vantage, plot type: XYZ 3D color and scale; different sizes in each follicular type denote different depths of follicles (smaller follicles are located the deeper from the surface). (**B**) Clustering analyses. Ovaries from animals at different ages were processed using Spot identification before analyzing clustering of follicles at the same developmental stage using the Ripley’s K function. The Y-axis depicts deviations of the follicle distribution (K(d)) from randomness (E(K(d)) over a given distance (X-axis) between center of follicles of the same size. Higher values indicate clustering whereas negative values indicate dispersion. (**C**) Follicle development from primary to antral stages was characterized by movement toward the center of the ovary. Distances from geometric center of the ovary to the center of individual follicles were determined in ovaries from mice at different ages (n = 3 ovaries). All values were normalized using location of the most peripheral primordial follicles (set at 1.0). Means + SE are shown.

We identified locations of follicles in ovaries from mice at different ages (Fig. 2A). Clustering of follicles at the same developmental stage was monitored by measuring spatial homogeneity using the Ripley’s K function that quantifies the degree of object clustering/dispersion as compared to a random distribution^17^. As shown in Fig. 2B, clustering indexes decreased as primordial follicles developed into primary, secondary and later stages in ovaries from animals at all ages. Of interest, there is also an age-dependent increase in follicle clustering from ovaries of day 10 to day 60 animals. We also analyzed distances of developing follicles from geometric center of the ovary using Imaris Spot and Surface algorithms. As shown in Fig. 2C, Spot plotting of distances of individual follicles from the center in adult ovaries suggested inward migration of primary, secondary, and antral follicles, consistent with earlier identification of primordial follicles near the periphery of ovaries in prepubertal and adult mice^6^.

### Analyses of follicles neighboring preovulatory follicles and corpora lutea

**Figure 3:**
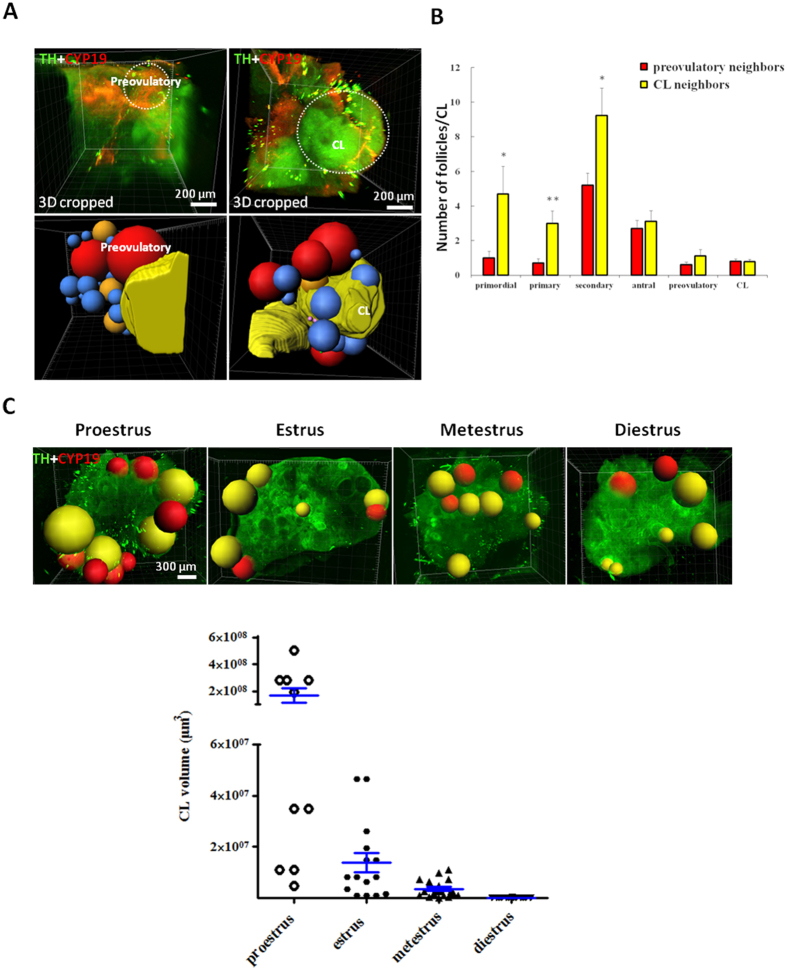
Analyses of follicles neighboring preovulatory follicles and corpora lutea. (**A**) Ovaries from proestrous mice were processed to reveal follicles in direct contact with preovulatory follicles (red color) and corpora lutea (CL, yellow color). Upper panel: 3D cropping of representative graphs showing selected preovulatory follicle and corpus luteum together with neighboring follicles. Lower panel: follicles shown as Spot graphs (red color: preovulatory; orange color: antral; blue color: secondary). Because preovulatory follicles and corpus lutea have irregular shapes, the Surface algorithm in Imaris was used to define their exact borders before neighboring analysis. Smaller and generally round follicles were reconstructed by using the Spot algorithm in Imaris. (**B**) Number of follicles neighboring individual preovulatory follicles or corpora lutea was plotted based on stages of follicle development (n = 10 from 5 ovaries). Stars indicate significant differences between numbers of follicles neighboring preovulatory follicles and CL (*P* < 0.05). Means + SE are shown. (**C**) Changes in volumes of corpora lutea during the estrous cycle. Upper panel: representative Spot graphs of corpora lutea (yellow color) and antral/preovulatory follicles (red color) at different stages of the estrous cycle. Lower panel: Decreases in volume of individual corpus luteum (CL) from proestrus to diestrus. Means ± SE are shown.

Three-D imaging analyses indicated that individual follicles were in close contact with other follicles or corpora lutea with minimal interstitial tissues. We digitally isolated individual preovulatory follicles and corpora lutea to estimate neighboring follicles in ovaries from proestrous mice (Fig. 3A). Corpora lutea were in direct contact with follicles of all stages whereas preovulatory follicles had fewer neighboring follicles. Quantitative analyses using ovaries from mice at different stages (Fig. 3B) indicated that preovulatory follicles had fewer neighboring primordial, primary, and secondary follicles as compared with those adjacent to the corpora lutea. In contrast, comparable numbers of antral and preovulatory follicles were found near preovulatory follicles and corpora lutea. By monitoring corpora lutea throughout all estrous stages by daily vaginal smears, we also found decreases in volumes of corpora lutea from proestrus to diestrus (Fig. 3C and D), suggesting rapid luteolysis within one reproductive cycle.

### Sphericity index analysis in follicles during development

**Figure 4:**
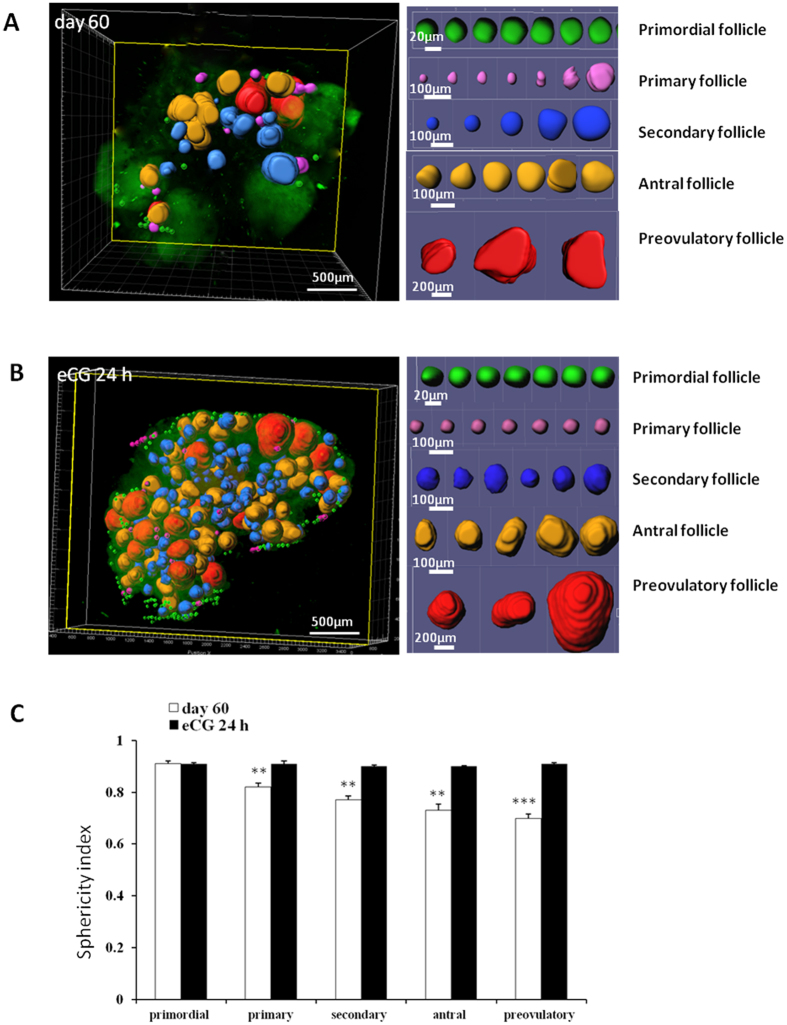
Decreases in sphericity of follicles during development. (**A**) A representative ovary from a proestrous mouse showing follicles at different developmental stages (left panel), together with gallery pictures of individual follicles arranged based on stages (right panel). (**B**) Same presentation of an ovary from an immature eCG-treated animal. (**C**) Sphericity indexes decreased with advancing stages of follicle development in ovaries of adult, but not eCG-treated immature mice. Stars indicate significant differences between different types of follicle and primordial follicle in each group (**P < 0.01, ***P < 0.01). Means + SE are shown.

We calculated sphericity of individual follicles. In ovaries of adult proestrous animals, roundness of follicles decreased as follicles developed into more advanced stages (Fig. 4A; left panel: whole ovary image; right panel: representative follicles at different stages). Unlike adult ovaries, no changes in sphericity were found during follicle development in ovaries of immature mice treated with equine chorionic gonadotropin (eCG) for 24 h (Fig. 4B and C), suggesting differences in ovarian rigidity. In adult, sphericity indices decreased from 0.91 in nearly round primordial follicles to 0.73 in irregularly shaped preovulatory follicles (Fig. 4C).

### Ovarian vasculature networks surrounding follicles and corpora lutea

**Figure 5:**
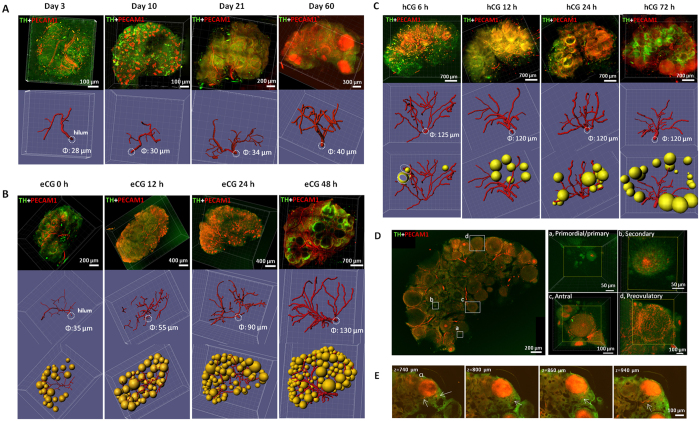
Ovarian vasculature networks surrounding follicles and corpora lutea. (**A**) Increases in vasculature during ovarian development. Ovaries from mice at different ages were immuno-stained for PECAM1 and TH to reveal vascular endothelial cells and follicles, respectively. Upper panels: 3D rendering of PECAM1 and TH staining. Lower panels: vasculature was traced using the Imaris Filament semi-manual algorithm. Diameters for vascular trunks originating from the helium (dashed cycle) are indicated. (**B**) Gonadotropin treatment increased ovarian vasculature. Immature mice at day 21 of age were treated with eCG for up to 2 days before immunostaining and imaging analyses. Upper panels: 3D rendering of PECAM1 and TH staining. Note non-specific PECAM1 staining in some oocytes of the eCG 24 h group. Middle panels: tracing of vasculature. Lower panels: Spot graphs of antral follicles situated near main vascular branches. Diameters for vascular trunks originating from the helium (dashed cycle) are indicated. (**C**) Luteinization of follicles was associated with massive neo-angiogenesis. Immature mice primed with eCG for 48 h were treated with hCG for up to 72 h to induce ovulation and luteinization of preovulatory follicles. Upper panels: 3D rendering of PECAM1 and TH staining. A time-dependent increase in PECAM1 staining signals was found in corpora lutea after hCG treatment. Middle panels: tracing of vasculature. Diameters for the main vascular trunks are shown. Lower panels: Spot graphs of luteinizing follicles situated near main vascular branches. At 6 h after hCG treatment, partially vascularized luteinizing follicles (outlined by dashed circles) showed local increases in PECAM1 staining (yellow areas). (**D**) One reprehensive ovary from immature mice treated with eCG for 24 h, together with enlarged areas showing (a) a lack of vasculature surrounding primordial/primary follicles and (b–d) increasing PECAM1 staining near thecal layers of secondary, antral, and preovulatory follicles. (**E**) Serial sections (740 to 940 um from surface) of a corpus luteum (CL) from the ovary of a metestrous mouse showing complete penetration of large blood vessels (arrows) into the structure.

We investigated ovarian vasculature changes following immunostaining for platelet-endothelial cell adhesion molecule 1 (PECAM1), a marker for endothelial cells^18^,^19^. PECAM1 staining in ovaries from mice at different ages was traced using the Imaris Filament algorithm and showed age-dependent increases in vasculature (Fig. 5A, upper panel: 3D rendering; lower panel: blood vessel tracing). In day 3 ovaries containing mainly primordial follicles, no PECAM1 staining outlining individual follicles were found despite the existence of major branches. In day 10 ovaries, growing secondary follicles showed strong staining in the theca layer whereas primordial/primary follicles in the cortical region had negligible PECAM1 signal. From day 3 to day 60 ovaries, major increases in vasculature branching were accompanied by a 40% increase in diameters of the largest vessel originating from the helium. Furthermore, gonadotropin treatment of immature mice led to time-dependent increases in ovarian vasculature (Fig. 5B). Diameters of the largest vessel showed a 3.7-fold increase at 48 h after gonadotropin treatment. After Spot transformation of individual follicles, a clear relationship between ovarian vasculature and follicles was found (Fig. 5B, lower panel). Following human chorionic gonadotropin (hCG) treatment of eCG-primed animals to induce ovulation, follicle luteinization was accompanied by rapid increases in vasculature inside preovulatory follicles and increases in volume of individual corpus luteum (Fig. 5C). In a representative adult ovary (Fig. 5D), PECAM1 staining in primordial/primary follicles was negligible whereas theca layers surrounding secondary, antral, and preovulatory follicles showed strong staining with no signal in inner granulosa cells. Serial sections of a representative corpus luteum (Fig. 5E) showed continuous PECAM1 staining throughout the solid structure. Supplementary Movie S3 showed relationship of blood vessels and antral/preovulatory follicles after eCG stimulation whereas Movie S4 showed invasion of PECAM1-stained endothelial cells into ruptured follicles during corpora lutea formation in hCG-treated animals. Movie S5 traced major vessels showing surrounding follicles in an ovary at 24 h after eCG treatment.

### Defective ovarian vasculature and ovulatory responses in VEGF promoter mutant mice

**Figure 6:**
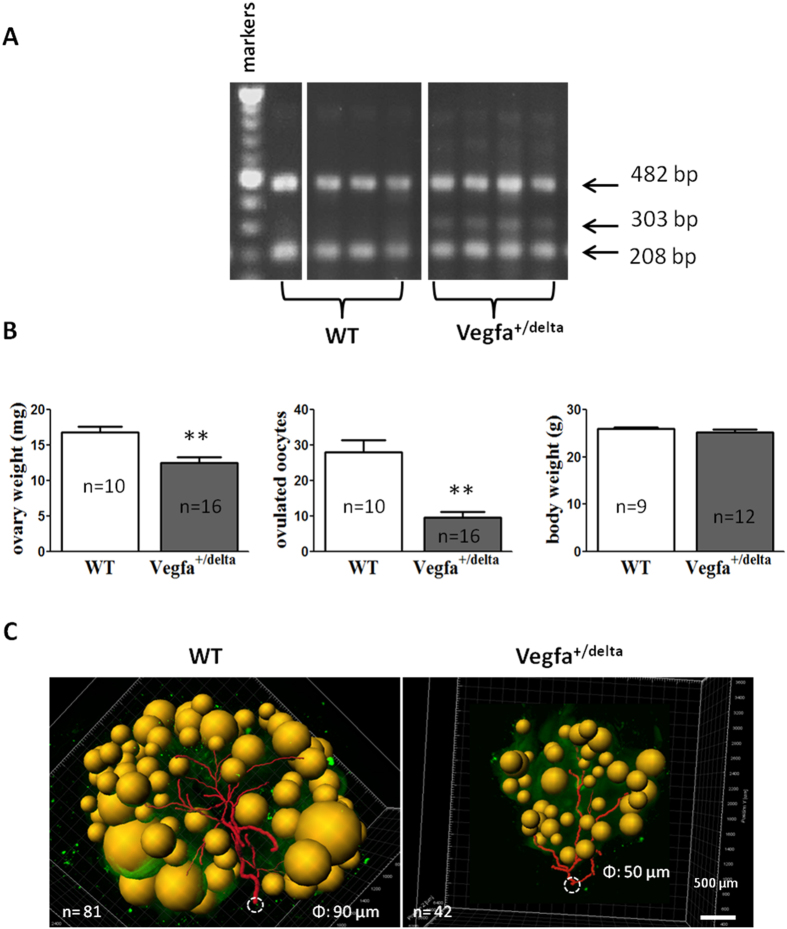
Defective ovarian vasculature and ovulatory responses in VEGFA mutant mice. (**A**) Wild type and heterozygous mutant *Vegfa*^+/delta^ mice with deletion of the hypoxia-response element in the VEGFA gene promoter were genotyped based on PCR analyses of tail DNA (cropped gel, full-length gel is included in Figure S4). (**B**) Wild type and heterozygous mice at 10–11 weeks of age were treated with eCG, followed by hCG to induce ovulation. Ovarian and body weights, together with numbers of ovulated oocytes were determined. WT: wild type. Stars indicate significant differences between wild type and *Vegfa*^+/delta^ mutant mice (*P* < 0.01). Means + SE are shown. (**C**) Tracing of ovarian vasculature in wild type and *Vegfa*^+/delta^ mutant mice. “N” represents number of total antral/preovulatory follicles in individual ovaries whereas Φ denotes the diameter of vascular trunks originating from the helium.

**Figure 7:**
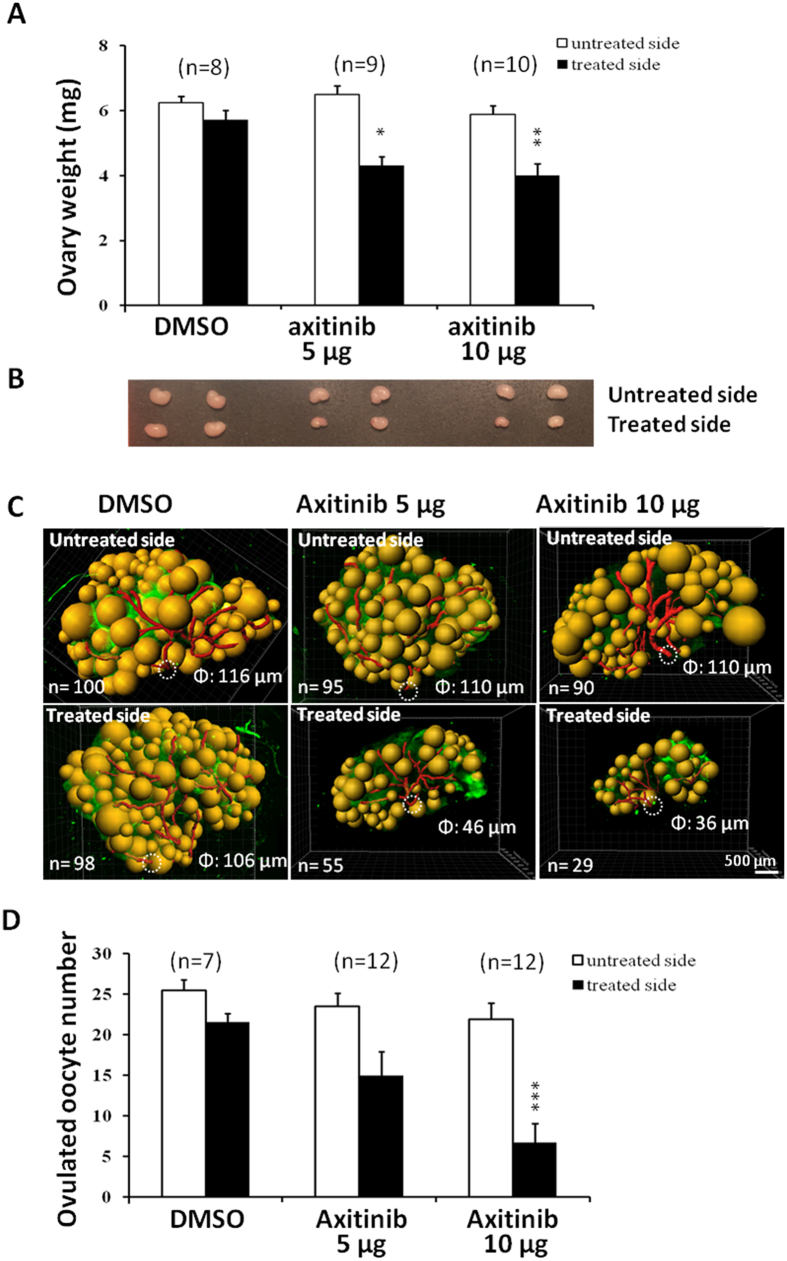
Unilateral intrabursal injection of a VEGF receptor inhibitor axitinib suppressed ovarian neo-angiogenesis, ovarian weight, and ovulation. Immature mice at 21 days of age were intrabursally injected with axitinib into the right ovary with the left side serving as controls. Control animals were injected unilaterally with the vehicle (DMSO). Animals were then treated with eCG to stimulate follicle growth. At 48 h after eCG injection, weights (**A**), morphology (**B**), and vasculature (**C**) of control and axitinib-treated ovaries were recorded. Stars indicate significant differences between untreated and treated sides (*P < 0.05 and **P < 0.05). Means + SE are shown. In (C),“n” represents number of total antral/preovulatory follicles in individual ovaries whereas Φ denotes the diameter of vascular trunks originating from the helium. (**D**) Some animals were further treated with hCG to induce ovulation before recording number of ovulated eggs retrieved. Stars indicate significant differences between untreated and treated sides (*P* < 0.001). Means + SE are shown.

To evaluate the importance of ovarian vasculature in ovulation and luteinization, we obtained *Vegfa*^delta/delta^ mutant mice with deletion of the hypoxia-response element in the VEGFA gene promoter^20^. These animals showed reduction of VEGF expression under hypoxia and defects in gonadotropin stimulation of VEGF expression^21^. After genotyping of wild type and heterozygous animals (Fig. 6A), we performed vaginal smearing of adult (~12 weeks of age) females and found heterozygous mutant animals were acyclic, showing prolonged diestrus. We then injected wild type and mutant animals at diestrus with eCG for 48 h, followed by hCG. At 16 h later, ovarian weights and number of ovulated oocytes were determined. As shown in Fig. 6B, mutant mice showed decreases in ovarian weights and lower number of oocytes released, together with a small decrease in body weight. CLARITY processing and immunostaining using PECAM1 antibodies indicated defective ovarian vasculature as shown by decreased total vasculature (Fig. 6C), presumably underlying the defective ovulatory responses. Furthermore, we uni-laterally injected axitinib, an inhibitor for all three VEGF receptor tyrosine kinases^22^, into ovarian bursa of immature wild type mice, before intraperitoneally treating the mice with eCG to induce follicle development. Forty-eight h later, axitinib pre-treatment decreased ovarian weights by ~30% (Fig. 7A and B) when compared with untreated contralateral ovaries in the same animal. Image analyses showed that axitinib treatment led to decreased total vasculature and lower antral/preovulatory follicle numbers (Fig. 7C). Following subsequent treatment with hCG to induce ovulation, axitinib-treated ovaries released fewer numbers of oocytes into oviducts (Fig. 7D).

## Discussion

Using the CLARITY approach, we performed marker staining, digital imaging, and spatial analysis to obtain 3D ovarian architecture throughout life. This method transforms intact tissues into a nanoporous hydrogel-hybridized form that is optically transparent and macromolecule-permeable^9^. Unlike classical 2D sectional histological analyses, CLARITY provides 3D mapping of interrelationships among follicles and corpora lutea at different developmental stages. Marker staining of ovarian structures revealed changes in follicle numbers, volume, clustering, migration, and sphericity, as well as previously unappreciated association among follicles and relationship between local vasculature and follicles/corpora lutea.

We found ~3 × 10^5^-fold increases in follicle volume from primordial to the preovulatory stage. This was accompanied by ~88,000-fold increases in oocyte volume, forming the largest cell in the body with a volume of 3 × 10^6^ um^3^. Spot transformation of 3D images of stained follicles facilitated quantitation of follicle numbers and confirmed age-dependent decreases of follicle numbers^5^. Findings of increases in percentages of primordial and primary follicles during senescence further suggested an age-related decline in follicle activation and development. Our findings related to age-dependent decline of number of primordial follicles are comparable to prior estimates of primordial follicle loss using a stereological method^23^ and whole mount immunofluorescence analyses^7^. For intact ovarian imaging, other methods such as X-ray imaging and ultrasound imaging have been developed in rodents^24^,^25^. Using a synchrotron X-ray imaging method, whole ovaries was evaluated to monitor Graffian and antral follicles at various ages in mice^26^. Although the exact age evaluated in that study were not comparable to our study, their findings showed lower number of antral follicles as observed here. Although X-ray and ultrasound imaging methods allowed imaging of antral follicles in the same animal overtime, these approaches do not allow evaluation of smaller follicles. On the other hand, traditional histological counting of follicles suffers from variability in section thickness, imprecise correction for total ovary volume, and a lack of consideration for follicle clustering, leading to large variations of follicle numbers differing up to ten-fold^27^. Using a whole-mount approach, a recent study minimized sectional errors and quantified oocyte dynamics in murine prenatal ovaries of small sizes^28^. The present approach using large adult ovaries provides a standardized way to determine follicle dynamics with minimal loss.

In ovaries from mice at different ages, we found clustering of follicles at same stages of development but clustering decreased as follicle development advanced. Earlier studies demonstrated clustering of preantral/early antral follicles in groups of up to 20–50 follicles^6^,^29^ whereas whole mount immunofluorescence analyses showed decreases in clustering as follicles develop based on nuclear staining in oocytes^7^. Highly clustered primordial follicles could secret follicle activation inhibitors as predicted by 2D spatial analysis^30^ whereas decreased clustering of secondary follicles is consistent with culture studies showing that two secondary follicles in direct contact invariably led to the dominance of one and growth suppression of its neighbor^31^. Consistent with rat studies^6^, small, non-growing primordial follicles were found under the surface epithelium in prepubertal and adult mice. Once primordial follicles are activated by local factors, they migrate away from the cortical region (Fig. 2C). This inward movement of follicles is likely due to the stiffer cortical enclosure as compared with the softer inner medulla. Although primordial and primary follicles are round in adult ovaries, sphericity indexes decreased in larger follicles. In contrast, advanced follicles maintained their roundness in ovaries of gonadotropin-treated immature mice. These data suggested that repeated tissue remodeling during recurring reproductive cycles could decrease tensile strength/elasticity in adult ovaries, rendering larger follicles into irregular shapes.

Our 3D imaging analyses indicated that follicles are in direct contact with each other. Although follicles at all stages were found adjacent to corpora lutea, preovulatory follicles were in close contact with antral, but fewer primordial, primary, and secondary follicles. These findings suggest that high levels of progesterone produced by the corpora lutea do not affect folliculogenesis whereas high levels of estrogen, inhibin, or other factors secreted by preovulatory follicles could confer unfavorable environments for preantral follicles. Also, we observed decreases in average volumes of corpora lutea from proestrous to the diestrous stage, suggesting rapid tissue remodeling and dissolution of the regressing structure within one estrous cycle.

In adult tissues, angiogenesis mainly occurs during wound healing and fracture whereas abnormal capillary growth is associated with pathological tumor growth, retinopathies, fibroses, and rheumatoid arthritis^32^. However, cyclic changes in ovarian and endometrial vasculature represent exceptions^4^,^8^. Consistent with the essential role of PECAM1 in vasculogenesis and angiogenesis^33^, we found major increase in PECAM1 staining during folliculogenesis and luteinization. Instead of randomly distributed inside the ovary, follicles are located along vascular branches. Growing follicles near vascular branches likely acquire more nutrients and secret more angiogenesis factors to promote local vasculature, resulting in a positive feedback loop. The adult ovary can be pictured as a vascular tree with 3 to 4 main branches each bearing fruits (follicles) of different sizes. Although the ovary does not have branching organelles similar to many other organs, intraovarian distribution of follicles is not a random event but is “constructed” around local vasculature.

We confirmed the avascular nature of cortical primordial follicles^8^. Because mTOR signaling regulated by nutrient availability is pivotal for the activation of dormant primordial follicles^34^,^35^, our data underscore the important role of local neo-angiogenesis in regulating follicle dormancy. In growing secondary and larger follicles, surrounding thecal cells have rich blood vessels that do not penetrate the basal membrane^32^. Extensive angiogenesis takes place following follicle rupture during luteinization and invasion of vasculature into corpora lutea is regulated by local pro- and anti-angiogenic factors^8^.

FSH stimulation of follicle growth is likely associated with hypoxia in the avascular granulosa cell compartment, leading to increased secretion of angiogenesis factors to promote neo-angiogenesis. The roles of ovarian VEGFA and neo-angiogenesis during gonadotropin induction of ovulation are demonstrated by our studies on ovulatory responses and vasculature changes in mutant mice with deletion of the hypoxia-response element in the VEGFA promoter. Because all three VEGF receptors are expressed in the ovary^36^,^37^,^38^, we unilaterally injected immature mice with axitinib, a pan-specific inhibitor for VEGF receptors, into ovarian bursa. Findings of defective ovarian vasculature and ovulatory responses after inhibitor treatment further underscore importance of endogenous VEGFs during gonadotropin-induced follicle maturation and ovulation.

Most ovarian research deals with molecular, cellular, and follicular scales, revealing roles of gonadotropins, paracrine factors, and local Hippo signaling^39^,^40^,^41^. The present approach facilitates studies at the whole ovary scale, highlighting the roles of vasculature, follicles interactions, and follicle locations in intact ovaries. The CLARITY approach provides opportunities to investigate hormonally regulated folliculogenesis, interrelationships among follicles and corpora lutea as well as remodeling of ovarian architecture during each reproductive cycle. Three-D imaging could allow elucidation of local tissue remodeling, including roles of invading macrophages during follicle rupture^42^ as well as fimbrial stem cells during surface epithelium repair^43^. CLARITY studies on cyclic ovarian remodeling confirm the theory that blood vessels direct the configuration of organs, first proposed by Aristotle^44^. Due to wide interests in using the CLARITY approach, recent modifications of this method has shortened the duration needed for tissue processing from weeks used here to days^45^. In addition, processing of larger samples is now possible^46^, thus allowing future evaluation of 3D architecture of the human ovary. In addition to providing models to investigate roles of neo-angiogenesis during organogenesis and tumorigenesis, future studies using the CLARITY approach could reveal ovarian architecture and vasculature underlying ovarian diseases, including polycystic ovarian syndrome, primary ovarian insufficiency, and ovarian tumorigenesis.

## Methods

### Study design

The experiments presented here were designed to investigate relationships among ovarian follicles, corpora lutea, and vasculatures as well as to test the hypothesis that ovarian vasculature dynamics are playing an important role in folliculogenesis and ovarian development. Using a newly established three-dimensional imaging technique, we provided evidences on vasculature control of folliculogenesis with the help of whole tissue imaging, together with immunostained markers in mouse ovaries. Furthermore, genetic and pharmacological mouse models were employed to regulate angiogenesis induced by VEGF signaling for evaluating folliculogenesis and ovulation. All experiments and analyses were repeated at least three times and were in accordance with the “Guide for the Care and Use of Laboratory Animals” (NIH publication 85–23, revised in 1996) and the “Principles of Laboratory Animal Care” (National Society for Medical Research). These experimental protocols and animal care were approved by regional committees (Stanford University, Project Nr. 10347 and Fudan University, Project Nr. 20150119-019).

### Animals and experimental design

Female C57BL/6 mice at different ages were obtained from Charles River Laboratories (Wilmington, MA). Aging C57BL/6 mice were from Shanghai Research Center for Model Organisms (Shanghai, China). Animals were housed in animal facilities at Stanford University or Fudan University under 12 h dark/light with free access to food and water. To investigate gonadotropin regulation of folliculogenesis, immature mice at 21 days of age were injected i.p. with 2 IU eCG (Peamex), followed at 48 h later by an injection of 10 IU hCG (Asuka Pharma) for up to 72 h. Animals were sacrificed at different times to obtain ovaries for CLARITY processing. To analyze atretic follicles, immature mice were treated with a single injection of 5 IU eCG for 96 h^47^. For studies using axitinib (Tocris), a pan-specific inhibitor for all three VEGF receptor tyrosine kinases^22^, mice at 21 days of age were anesthetized using isoflurane (Piramal Group) and injected with axitinib (5 or 10 μg dissolved in 10 μl DMSO; Tocris Inc.) using a 31-gauge insulin syringe (BD Inc.) through the ovarian fat pad into the bursa of the right ovary. This was followed by an injection of 5 IU eCG to stimulate follicle development, before collection of ovaries at 48 h after eCG treatment for weighing and CLARITY processing. To estimate ovulatory potency, animals were injected with 10 IU hCG at 48 h after eCG treatment before counting numbers of oocytes in oviducts at 16 h later. Each group included 6 to 12 mice.

### Genotyping and ovulation induction of mutant mice

*Vegfa*^delta/delta^ mice were generated by mating male and female heterozygous animals for the transgenic allele and maintained on a C57BL/6 genetic background. Wild type C57BL/6 mice were obtained from the Charles River Laboratory. Genotyping analyses were performed by PCR on DNA obtained from tail biopsies using the oligonucleotide primers 5′-GCTTATCTGAGCCCTTGTCTGATC-3′ (*Vegfa* genotype sense probe); 5′-GATGAACCGTAAGCCTAGGCTAG-3′ (*Vegfa* genotype antisense probe); 5′-GAGTGAGACGACCTGTGGAAATG-3′ (mutant-sense probe); and 5′-AGACTACACAGTGCATACGTGGGT-3′ (wild type-antisense probe) based on the following PCR conditions: 1 min at 94 °C for one cycle, 30 sec at 94 °C, 30 sec at 60 °C, and 30 sec at 72 °C for 29 cycles, and 10 min at 72 °C for one cycle. Primers were designed to generate PCR products of 482 bp and 208 bp for the wild type *Vegfa* allele, or 482, 303, and 208 bp for the heterozygous *Vegfa*^+/delta^ allele. Adult (10–11 weeks of age) wild type and heterozygous mutant mice were injected i. p. with 7.5 IU of eCG on the diestrous day, followed by a single injection of hCG (10IU) at 48 h later. At 16 h after hCG injection, ovarian and body weights were determined together with counting the number of ovulated oocytes in oviducts to estimate ovulatory potential. Some ovaries were further processed using CLARITY and immunostaining to evaluate ovarian vasculature.

### CLARITY processing of ovaries

Mice were anesthetized i.p. with 2, 2, 2-Tribromoethanol (250 mg/kg body weight, Sigma-Aldrich) before transcardially perfused with 10 (postnatal day 3 or 10) or 20 ml (postnatal day 21 or older) of ice-cold 1 × PBS (Invitrogen GIBCO), followed by 10 or 20 ml of ice-cold hydrogel solution with 4% (weight/volume) paraformaldehyde (Electron Microscopy Sciences), 4% acrylamide (Bio-Rad), 0.05% bis-acrylamide (Bio-Rad), 0.25% VA-044 (Wako), 0.05% Saponin (Sigma) and 1 × PBS for 2 min as described^9^. Fixed ovaries were immediately extracted and immersed in 10 ml conical tubes containing 6–7 ml of the hydrogel solution for 3–4 days at 4 °C. Then, tubes were de-gassed with dry ice to remove oxygen and submerged in a 37 °C water bath for 3 h to polymerize hydrogel monomers. Embedded samples were then transferred into 10 ml of the clearing solution (200 mM boric acid (Sigma), 4% (wt/vol) sodium dodecyl sulfate (SDS) (Sigma) dissolved in distilled H_2_O, with the addition of NaOH (EMD) to reach pH 8.5^9^. The clearing solution was replaced 2–3 times/week and ovaries gradually became transparent after 4–8 weeks depending their sizes and ages of the animal. We performed CLARITY without the electrophoretic step to allow better antibody staining and to minimize damage of small and fragile murine ovaries.

### Immunofluorescence staining

Cleared ovaries were transferred into 10 ml glass bottles containing PBST (PBS with 0.1% Triton-X (Sigma)) before shaking slowly in PBS for 1 day to remove residual SDS. Samples were then incubated with primary antibodies for two days, washed in buffer for 1 day, followed by secondary antibodies for 2 days. Before mounting and imaging, samples were incubated in PBST for at least 1 day in the FocusClear solution (CelExplore, Taiwan) for 1 h to correct for refractive index. All procedures were conducted with shaking at 37 °C. Detailed antibody information is shown in Table S3.

### Digital imaging and data analysis

Intact ovaries were mounted on glass slides using MountClear (CelExplore, Taiwan) and Willco-Dish (Ted Pella, CA). Images were obtained using Leica SP5 confocal microscope (20 × water-immersion objectives; numerical aperture 1.0; working distance 2.0 mm) and Olympus confocal microscopes (25 × water-immersion objectives; numerical aperture 0.95; working distance 2.5 mm; single-photon excitation). Ovary scanning was taken at the maximum resolution using the Leica tandem resonant scanner (image resolution 1024 × 1024 pixels) and images were stored in the TIFF format. Two-D and 3D imaging as well as data analysis were performed using the Fiji ImageJ and Imaris software (V 8.0, Bitplane, Switzerland). Spot transformation was based on the Imaris algorithm. Because imperfection of staining, some follicles were identified semi-manually using the Spot algorithm in the Imaris software (Figure S3). Spatial homogeneity was determined using Ripley’s K function for point pattern analysis by quantifying the degree of object clustering/dispersion as compared to a random distribution^17^. Roundness of follicles was determined using Imaris Surface algorithm semi-manually. After defining follicle borders according to signals in the related sections, the software automatically made an object that was measurable by the statistic option of the Imaris software. Whole mount analysis of branching structures was generated semi-manually based on Filament algorithms developed for studies on kidney glomeruli^49^. After setting the starting point of the blood vessel, the software detected signals to make a continuous line along the vessel. By setting the signal thresholds in the software, the cylinder shape of the vessels could be formed. To finalize the 3D construction of vessels with the Filament option, data related to the designed branching structures were measured by using the Imaris software.

### Statistical analysis

All data were presented as standard errors of mean (SE). Kolmogorov-Smirnov test was performed for evaluation of the data normality. For multiple groups, statistical analysis was done with independent sample t-test or one-way ANOVA and Tukey *post-hoc* test (SPSS for Windows, version 20, SPSS Inc, Chicago, Illinois, USA). The *P* values of less than 0.05 referred to statistically significance.

## Additional Information

**How to cite this article:** Feng, Y. *et al*. CLARITY reveals dynamics of ovarian follicular architecture and vasculature in three-dimensions. *Sci. Rep.* **7**, 44810; doi: 10.1038/srep44810 (2017).

**Publisher's note:** Springer Nature remains neutral with regard to jurisdictional claims in published maps and institutional affiliations.

## Supplementary Material

Supplementary Information

Supplementary Video S1

Supplementary Video S2

Supplementary Video S3

Supplementary Video S4

Supplementary Video S5

## References

[b1] Deepak A. & Kui L. Molecular mechanisms underlying the activation of mammalian primordial follicles. Endocr. Rev. 30, 438–464 (2009). 19589950 10.1210/er.2008-0048

[b2] McGee E. A. & Hsueh A. J. Initial and cyclic recruitment of ovarian follicles. Endocr. Rev. 21, 200–214 (2000). 10782364 10.1210/edrv.21.2.0394

[b3] Macklon N. S., Stouffer R. L., Giudice L. C. & Fauser B. C. The science behind 25 years of ovarian stimulation for *in vitro* fertilization. Endocr. Rev. 27, 170–207 (2006). 16434510 10.1210/er.2005-0015

[b4] Hazzard T. M. & Stouffer R. L. Angiogenesis in ovarian follicular and luteal development. Baillieres Best Pract. Res. Clin. Obstet. Gynaecol. 14, 883–900 (2000). 11141339 10.1053/beog.2000.0133

[b5] Faddy M., Gosden R., Gougeon A., Richardson S. J. & Nelson J. Accelerated disappearance of ovarian follicles in mid-life: implications for forecasting menopause. Hum. Reprod. 7, 1342–1346 (1992). 1291557 10.1093/oxfordjournals.humrep.a137570

[b6] Hirshfield A. N. & DeSanti A. M. Patterns of ovarian cell proliferation in rats during the embryonic period and the first three weeks postpartum. Biol. Reprod. 53, 1208–1221 (1995). 8527527 10.1095/biolreprod53.5.1208

[b7] Faire M. . Follicle dynamics and global organization in the intact mouse ovary. Dev. Biol. 403, 69–79 (2015). 25889274 10.1016/j.ydbio.2015.04.006 PMC4469539

[b8] Fraser H. M. Regulation of the ovarian follicular vasculature. Reprod. Biol. Endocrinol. 4, 18 (2006). 16611363 10.1186/1477-7827-4-18 PMC1459163

[b9] Chung K. . Structural and molecular interrogation of intact biological systems. Nature 497, 332–337 (2013). 23575631 10.1038/nature12107 PMC4092167

[b10] Menezes J. & Luskin M. Expression of neuron-specific tubulin defines a novel population in the proliferative layers of the developing telencephalon. J. Neurosci. 14, 5399–5416 (1994). 8083744 10.1523/JNEUROSCI.14-09-05399.1994 PMC6577108

[b11] D’Albora H., Lombide P. & Ojeda S. R. Intrinsic neurons in the rat ovary: an immunohistochemical study. Cell Tissue Res. 300, 47–56 (2000). 10805074 10.1007/s004419900130

[b12] Dominguez M. A., Cho N., Zhang B., Neal M. S. & Foster W. G. Brain-derived neurotrophic factor expression in granulosa lutein cells. Reprod. Biomed. Online 22, 17–24 (2011). 21115268 10.1016/j.rbmo.2010.09.001

[b13] Weenen C. . Anti-Müllerian hormone expression pattern in the human ovary: potential implications for initial and cyclic follicle recruitment. Mol. Hum. Reprod. 10, 77–83 (2004). 14742691 10.1093/molehr/gah015

[b14] Sai T. . Bid and Bax are involved in granulosa cell apoptosis during follicular atresia in porcine ovaries. J. Reprod. Dev. 57, 421–427 (2011). 21441714 10.1262/jrd.11-007h

[b15] Hsueh A. J., Adashi E. Y., Jones P. B. & Welsh T. H. Jr. Hormonal regulation of the differentiation of cultured ovarian granulosa cells. Endocr. Rev. 5, 76–127 (1984). 6142819 10.1210/edrv-5-1-76

[b16] Faddy M., Gosden R. & Edwards R. Ovarian follicle dynamics in mice: a comparative study of three inbred strains and an F1 hybrid. J. Endocrinol. 96, 23–33 (1983). 6822780 10.1677/joe.0.0960023

[b17] Ripley B. D. Modelling spatial patterns. J. Roy. Stat. Soc. Ser. B. 172–212 (1977).

[b18] Cao G. . Angiogenesis in platelet endothelial cell adhesion molecule-1-null mice. Am. J. Pathol. 175, 903–915 (2009). 19574426 10.2353/ajpath.2009.090206 PMC2716984

[b19] Newman P. J. The biology of PECAM-1. J. Clin. Invest. 99, 3 (1997). 9011572 10.1172/JCI119129 PMC507759

[b20] Oosthuyse B. . Deletion of the hypoxia-response element in the vascular endothelial growth factor promoter causes motor neuron degeneration. Nat. Genet. 28, 131–138 (2001). 11381259 10.1038/88842

[b21] Rico C. . HIF1 Activity in Granulosa Cells Is Required for FSH-Regulated Vegfa Expression and Follicle Survival in Mice 1. Biol. Reprod. 90, 131–137 (2014). 24855100 10.1095/biolreprod.113.115634

[b22] Hu-Lowe D. D. . Nonclinical antiangiogenesis and antitumor activities of axitinib (AG-013736), an oral, potent, and selective inhibitor of vascular endothelial growth factor receptor tyrosine kinases 1, 2, 3. Clin. Cancer Res. 14, 7272–7283 (2008). 19010843 10.1158/1078-0432.CCR-08-0652

[b23] Kerr J. B., Myers M. & Anderson R. A. The dynamics of the primordial follicle reserve. Reproduction, 146, R205–R215 (2013). 23929903 10.1530/REP-13-0181

[b24] Mircea C. N. . Ovarian imaging in the mouse using ultrasound biomicroscopy (UBM): a validation study. Reprod. Fertil. Develop. 21, 579–586 (2009). 10.1071/RD08295 PMC2882435 19383264

[b25] Jaiswal R. S., Singh J. & Adams G. P. High-resolution ultrasound biomicroscopy for monitoring ovarian structures in mice. Reprod. Biol. Endocrinol. 7, 69 (2009). 19580664 10.1186/1477-7827-7-69 PMC2714516

[b26] Kim J., Choi Y. H., Chang S., Kim K.-T. & Je J. H. Defective folliculogenesis in female mice lacking Vaccinia-related kinase 1. Sci. Rep. 2, 468 (2012). 22741057 10.1038/srep00468 PMC3384087

[b27] Myers M., Britt K. L., Wreford N. G., Ebling F. J. & Kerr J. B. Methods for quantifying follicular numbers within the mouse ovary. Reproduction 127, 569–580 (2004). 15129012 10.1530/rep.1.00095

[b28] Malki S., Tharp M. E. & Bortvin A. A whole-mount approach for accurate quantitative and spatial assessment of fetal oocyte dynamics in mice. Biol. Reprod. 93, 113 (2015). 26423126 10.1095/biolreprod.115.132118

[b29] Baker S. J. & Spears N. The role of intra-ovarian interactions in the regulation of follicle dominance. Hum. Reprod. Update 5, 153–165 (1999). 10336019 10.1093/humupd/5.2.153

[b30] Da Silva-Buttkus P., Marcelli G., Franks S., Stark J. & Hardy K. Inferring biological mechanisms from spatial analysis: prediction of a local inhibitor in the ovary. Proc. Natl. Acad. Sci. USA 106, 456–461 (2009). 19122142 10.1073/pnas.0810012106 PMC2626724

[b31] Spears N., de Bruin J. P. & Gosden R. G. The establishment of follicular dominance in co-cultured mouse ovarian follicles. J. Reprod. Fertil. 106, 1–6 (1996). 8667332 10.1530/jrf.0.1060001

[b32] Reynolds L. P., Killilea S. & Redmer D. Angiogenesis in the female reproductive system. FASEB J. 6, 886–892 (1992). 1371260

[b33] Duncan G. S. . Genetic evidence for functional redundancy of platelet/endothelial cell adhesion molecule-1 (PECAM-1): CD31-deficient mice reveal PECAM-1-dependent and PECAM-1-independent functions. J. Immunol. 162, 3022–3030 (1999). 10072554

[b34] Zhang H. . Somatic cells initiate primordial follicle activation and govern the development of dormant oocytes in mice. Curr. Biol. 24, 2501–2508 (2014). 25438940 10.1016/j.cub.2014.09.023

[b35] Laplante M. & Sabatini D. M. mTOR signaling in growth control and disease. Cell 149, 274–293 (2012). 22500797 10.1016/j.cell.2012.03.017 PMC3331679

[b36] Pauli S. A. . The vascular endothelial growth factor (VEGF)/VEGF receptor 2 pathway is critical for blood vessel survival in corpora lutea of pregnancy in the rodent. Endocrinology 146, 1301–1311 (2005). 15591152 10.1210/en.2004-0765

[b37] Rutkowski J. M. . VEGFR-3 neutralization inhibits ovarian lymphangiogenesis, follicle maturation, and murine pregnancy. Am. J. Pathol. 183, 1596–1607 (2013). 24036251 10.1016/j.ajpath.2013.07.031 PMC3814520

[b38] Endo T. . Cyclic changes in expression of mRNA of vascular endothelial growth factor, its receptors Flt-1 and KDR/Flk-1, and Ets-1 in human corpora lutea. Fertil. Steril 76, 762–768 (2001). 11591411 10.1016/s0015-0282(01)02012-x

[b39] Zeleznik A. J. Follicle selection in primates: “many are called but few are chosen”. Biol. Reprod. 65, 655–659 (2001). 11514325 10.1095/biolreprod65.3.655

[b40] Macklon N. S. & Fauser B. C. Aspects of ovarian follicle development throughout life. Horm. Res. 52, 161–170 (1999). 10725781 10.1159/000023456

[b41] Hsueh A. J., Kawamura K., Cheng Y. & Fauser B. C. Intraovarian control of early folliculogenesis. Endocr. Rev. 36, 1–24 (2015). 25202833 10.1210/er.2014-1020 PMC4309737

[b42] Wu R., Van der Hoek K. H., Ryan N. K., Norman R. J. & Robker R. L. Macrophage contributions to ovarian function. Hum. Reprod. Update 10, 119–133 (2004). 15073142 10.1093/humupd/dmh011

[b43] Ng A. & Barker N. Ovary and fimbrial stem cells: biology, niche and cancer origins. Nat. Rev. Mol. Cell Biol. 16, 625–638 (2015). 26350076 10.1038/nrm4056

[b44] Crivellato E., Nico B. & Ribatti D. Contribution of endothelial cells to organogenesis: a modern reappraisal of an old Aristotelian concept. J. Anat. 211, 415–427 (2007). 17683480 10.1111/j.1469-7580.2007.00790.x PMC2375830

[b45] Treweek J. B. . Whole-body tissue stabilization and selective extractions via tissue-hydrogel hybrids for high-resolution intact circuit mapping and phenotyping. Nat. Proto. 10, 1860–1896 (2015). 10.1038/nprot.2015.122 PMC4917295 26492141

[b46] Woo J., Lee M., Seo J. M., Park H. S. & Cho Y. E. Optimization of the optical transparency of rodent tissues by modified PACT-based passive clearing. Exp. Mol. Med. 48, e274 (2016). 27909337 10.1038/emm.2016.105 PMC5192069

[b47] Hsueh A. J., Billig H. & Tsafriri A. Ovarian follicle atresia: a hormonally controlled apoptotic process. Endocr. Rev. 15, 707–724 (1994). 7705278 10.1210/edrv-15-6-707

[b48] Tomer R., Ye L., Hsueh B. & Deisseroth K. Advanced CLARITY for rapid and high-resolution imaging of intact tissues. Nat. Prot. 9, 1682–1697 (2014). 10.1038/nprot.2014.123 PMC4096681 24945384

[b49] Short K. M. . Global quantification of tissue dynamics in the developing mouse kidney. Dev. Cell 29, 188–202 (2014). 24780737 10.1016/j.devcel.2014.02.017

