# Supplementary material for: CLARITY reveals dynamics of ovarian follicular architecture and vasculature in three-dimensions
Source: Sci Rep. 2017 Mar 23;7:44810. doi: 10.1038/srep44810 (PMC5363086; doi:10.1038/srep44810)
Supplement: Supplementary Information [file srep44810-s1.pdf]

CLARITY reveals dynamics of ovarian follicular architecture and vasculature in three-dimensions

Yi Feng, Peng Cui, Xiaowei Lu, Brian Hsueh, Fredrik Möller Billig, Livia Zarnescu Yanez, Raju Tomer, Derek Boerboom, Peter Carmeliet, Karl Deisseroth, and Aaron J. W. Hsueh

SUPPLEMENTARY MATERIALS

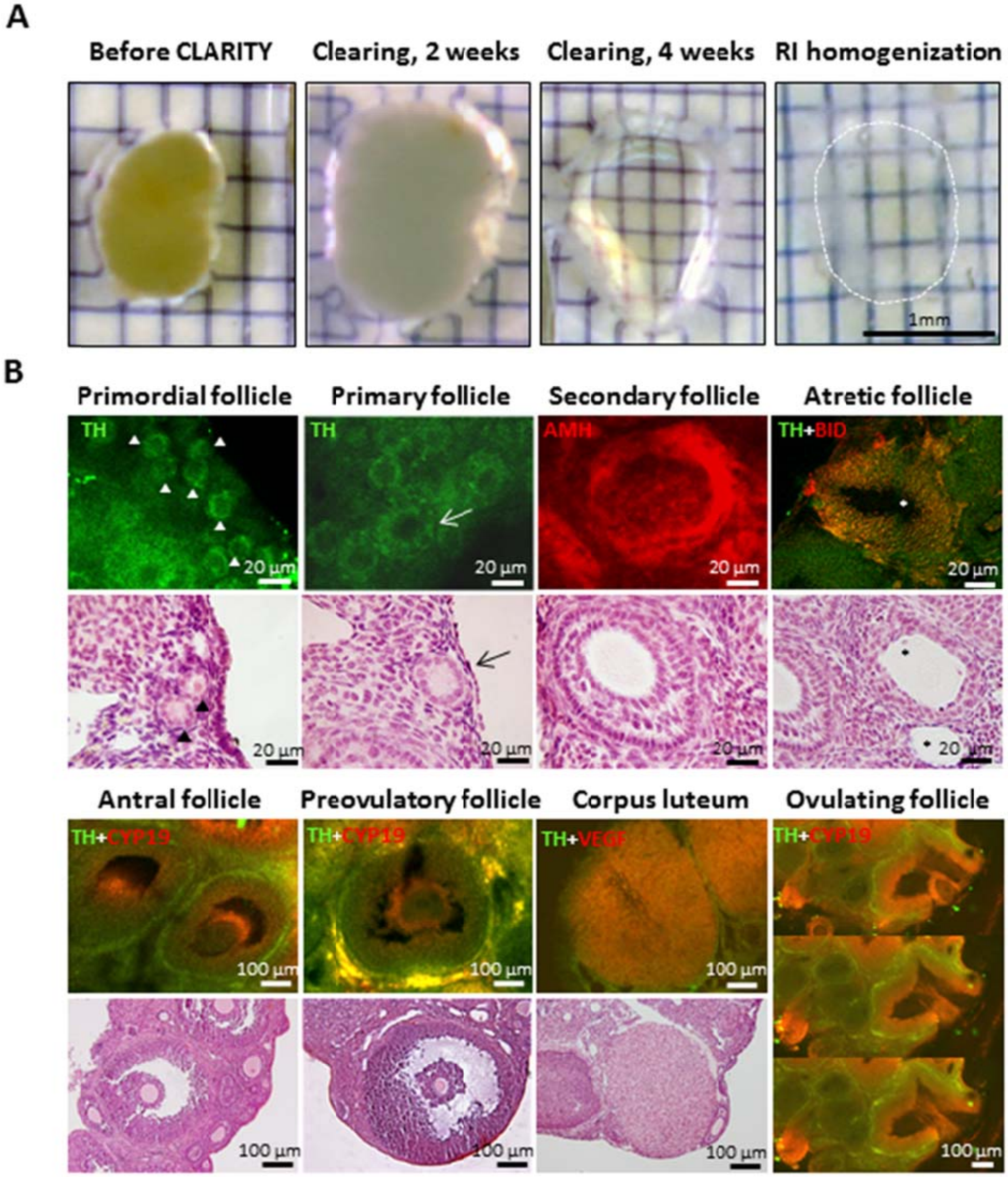

Figure S1. Identification of ovarian follicles and corpora lutea at different developmental stages using specific markers. (**A**) Processing of an ovary using CLARITY. An ovary from an adult proestrous mouse was processed using the CLARITY method, followed by incubation in the clearing buffer for 4 weeks before immunostaining. After staining, samples were incubated

for 1h at 37°C in the FocusClear medium for reflective index (RI) homogenization. Although initial clearing led to tissue shrinkage, subsequent RI homogenization restored the original size. (**B**) Staining of follicles at different stages using specific markers (upper panels) together with histological pictures using hematoxylin and eosin staining (lower panels). Pictures for primordial, primary, and secondary follicles were from ovaries of day 10 mice whereas those for antral, preovulatory, and rupturing follicles together with corpus luteum were from adult proestrous mice. Atretic follicles were from ovaries of immature mice following gonadotropin treatment and then withdrawal. “Ovulating follicle” denotes serial sections of a follicle during rupture. TH, tyrosine hydroxylase; AMH, anti-Müllerian hormone; BID, BH3-interacting domain death agonist; CYP19, aromatase; VEGF, vascular endothelial growth factor. Arrows and arrowheads denote individual follicles whereas asterisks indicate follicle antrum.

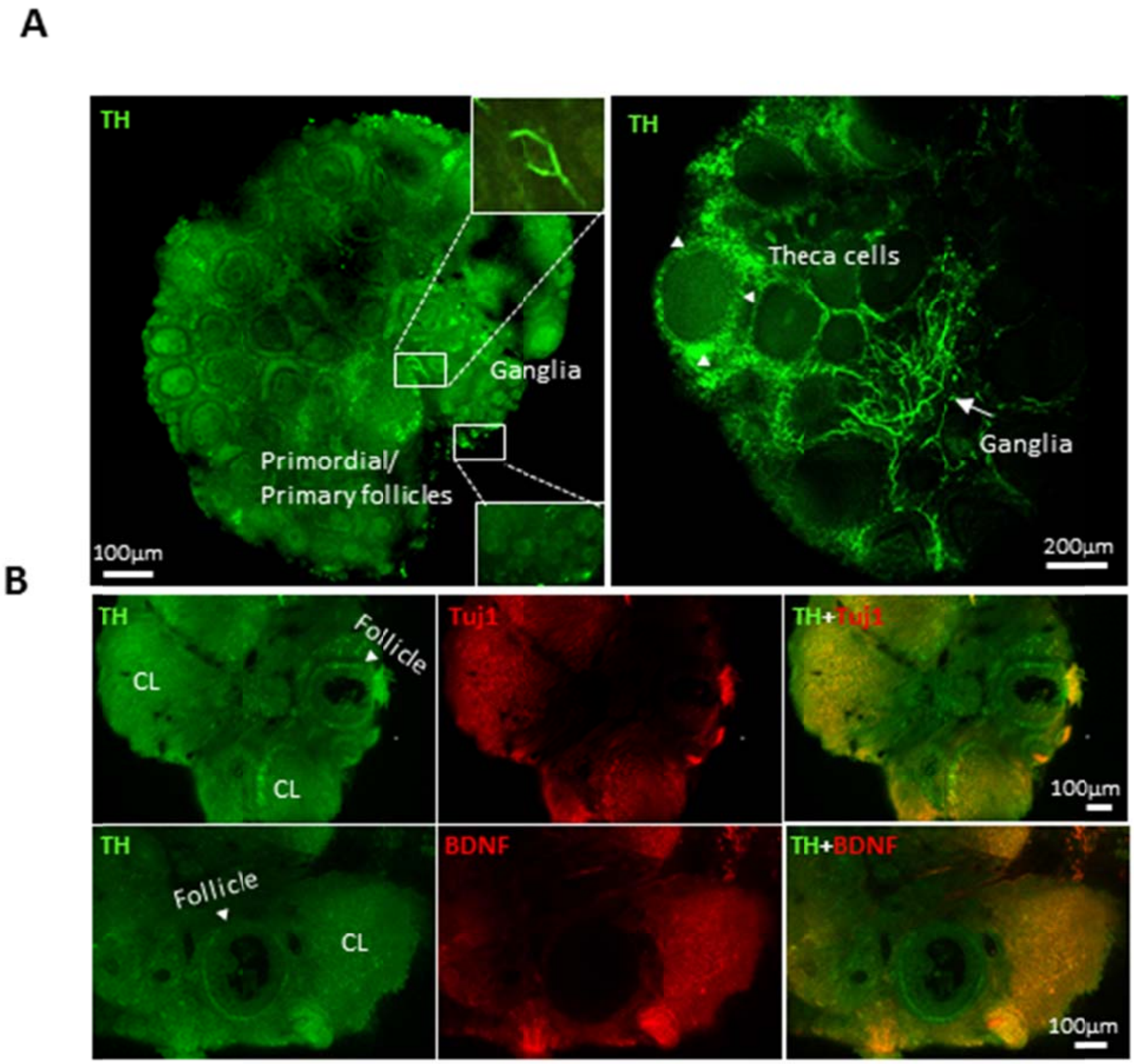

Figure S2. Ovarian staining in theca cells, ganglia, and corpora lutea (CL) using tyrosine hydroxylase (TH) antibodies. Ovaries from day 10 (**A** right panel) and adult mice (**A** right panel and **B**) were used for Clarity processing and immunostaining for TH, neuron-specific class III beta-tubulin (Tuj1), and brain-derived neurotropic factor (BDNF). TH staining was evident in theca layer surrounding secondary follicles, ganglia, and CL. Co-staining for TH and Tuj1 or BDNF was found in theca cells and CL. Note nonspecific background staining of TH in all follicles.

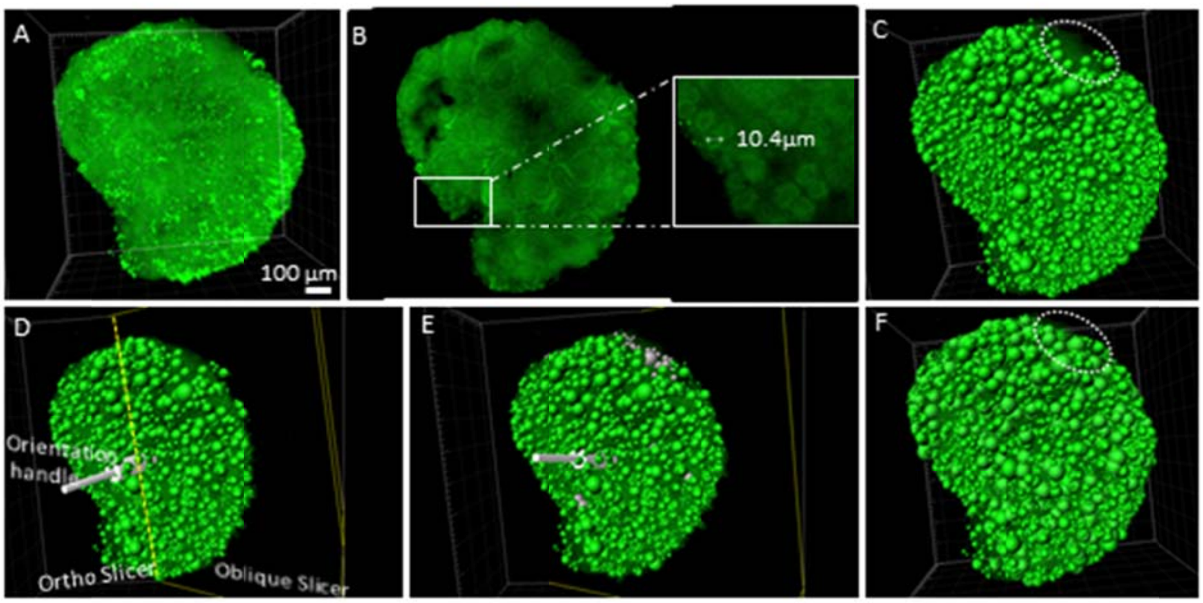

Figure S3. Spot identification and manual improvement. Follicle numbers were calculated using Imaris analysis following Clarity and immunostaining for tyrosine hydroxylase. (**A**) 3D imaging was obtained using confocal microscope after adjusting optimal channel display. (**B**) The Slice function in Imaris was used to look through each frame of the entire ovary and estimate minimal diameters of follicles. The boxed area was amplified to indicate small follicles at 10.4 µm diameter. (**C**) Spot automatic algorithm in Imaris was used to calculate follicular volumes by checking for spot sizes, designating source channels, estimating diameters (>10.4 µm) and subtracting background noises, followed by adjustment of quality parameters and spot regions of interest. Accuracy of spot determination depended on the quality of staining and image resolution. The dotted circle shows missing spots due to low signals. (**D**) The Oblique Slicer and Ortho Slicer functions in Imaris were used to manually improve spot signals. (**E**) Slice position and Slice orientation (XY, XZ, and YZ planes) were used to check accuracy for individual follicle prediction. Software could suggest adding/deleting spots. (**F**) Merging all spots together allows determination of follicle numbers, diameters, volumes and positions. The dotted circle shows amended spots.

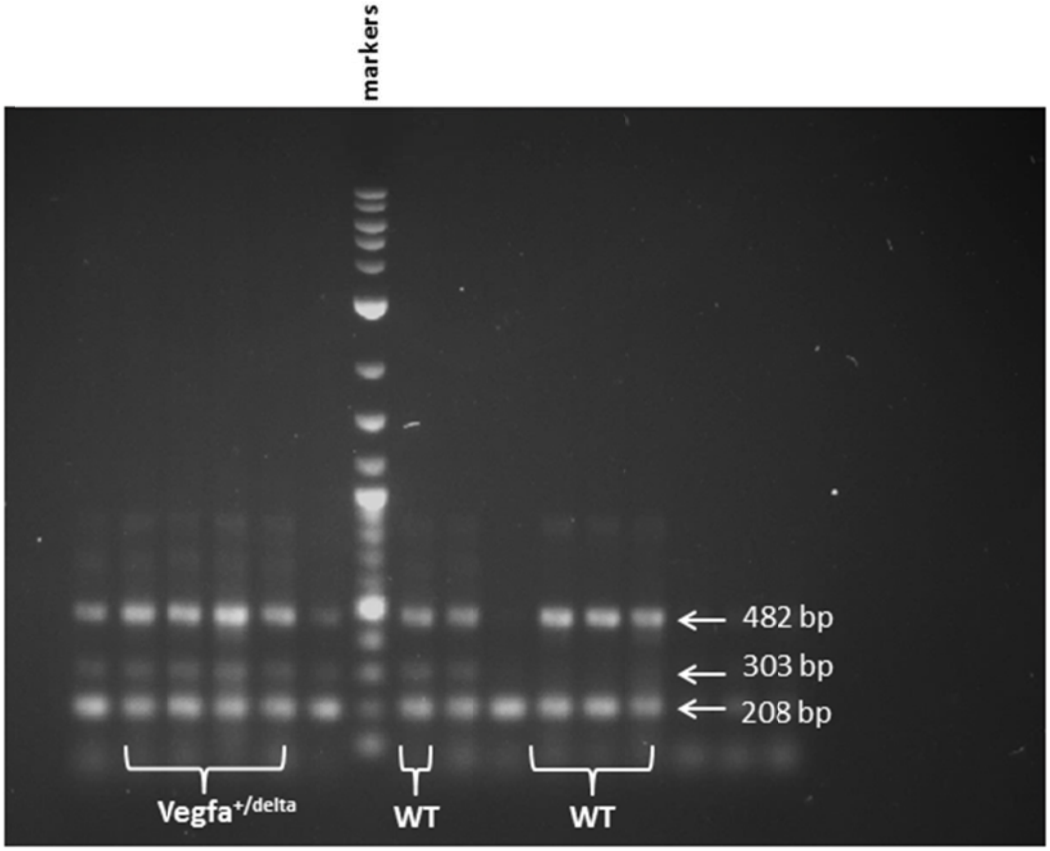

Figure S4. Full-length gel related to the cropped gel of Figure 6A. Wild type and heterozygous mutant *Vegfa*$^{+/delta}$ mice with deletion of the hypoxia-response element in the VEGFA gene promoter were genotyped based on PCR analyses of tail DNA.

Movies

Movie S1. Three-dimensional images of a day-10 mouse ovary were generated for Spot transformation, followed by manual improvement, to identify all follicles.

Movie S2. Construction of follicle relationship maps using specific markers in ovary of an adult proestrous mouse. Follicles at all developmental stages were traced together with corpora lutea in the ovary.

Movie S3. Relationship of blood vessels and antral/preovulatory follicles after equine chorionic gonadotropin (eCG) stimulation.

Movie S4. Corpora lutea and vasculature networks in mice ovaries treated with human chorionic gonadotropin (hCG) and equine chorionic gonadotropin (eCG) for different duration. PECAM1, platelet-endothelial cell adhesion molecule 1; TH, tyrosine hydroxylase.

Movie S5. Ovarian vasculature surrounding follicles in an ovary at 24h after equine chorionic gonadotropin (eCG) treatment. PECAM1, platelet-endothelial cell adhesion molecule 1; TH, tyrosine hydroxylase.

Table S1. Parameters (diameter, surface area, and volume) for follicles, oocytes, and corpora lutea at different developmental stages in mice.

Ovarian structure	Diameter (μm)	Surface area (μm^2)	Volume (μm^3)
Follicle			
Primordial	7-25	154-1,963	180-8,181
Primary	25-55	1,963-9,503	8,181-87,114
Secondary	50-250	$0.078\text{-}2\times10^5$	$0.65\text{-}82\times10^5$
Antral	200-350	$1\text{-}4\times10^5$	$42\text{-}225\times10^5$
Preovulatory	350-600	$9\text{-}11\times10^5$	$449\text{-}626\times10^5$
Oocyte			
Primordial follicle	4-6	50-113	34-113
Primary follicle	8-30	200-380	268-697
Secondary follicle	13-65	531-13,273	$0.01\text{-}1.4\times10^5$
Antral follicle	80-120	20,106-45,239	$2.7\text{-}9\times10^5$
Preovulatory follicle	85-180	$0.23\text{-}1\times10^5$	$3\text{-}30\times10^5$
Corpus luteum	250-850	$2\text{-}59\times10^5$	$70\text{-}5{,}093\times10^5$

Table S2. Fold changes of different parameters for follicles at different developmental stages in mice.

Ovarian structure	Diameter (μm)	Surface area（$μm^2$）	Volume ($μm^3$)
Primordial follicle	3.6	12.75	45.00
primary follicle	2.2	4.84	10.65
Secondary follicle	5	25.00	125.00
Antral follicle	1.75	3.00	5.36
Preovulatory follicle	1.71	1.27	1.39
Corpus luteum	3.4	28.00	73.09

Table S3. Antibodies information.

Antibodies	Species	Dilution	Company	Cat. No	Markers for
Primary antibodies					
TH (tyrosine hydroxylase)	Rabbit	1:50	Abcam	ab112	Neurons
TH (tyrosine hydroxylase)	Chicken	1:50	Abcam	ab76442	Neurons
Tuj1 (neuron-specific class III beta-tubulin)	Rabbit	1:50	Abcam	ab18207	Neurons
BDNF (brain-derived neurotropic factor)	Rabbit	1:50	Abcam	ab72439	Neurons
PECAM1 (platelet-endothelial cell adhesion molecule 1)	Rabbit	1:10	Abcam	ab28364	Blood vessel (endothelium cells)/corpus luteum
AMH (anti-Müllerian hormone)	Goat	1:50	Santa Cruz	sc-6886	Granulosa cell (secondary follicle)
CYP19 (aromatase)	Rabbit	1:50	Abcam	ab35604	Granulosa cell (antral and preovulatory follicle)
Bid (BH interacting domain death agonist)	Goat	1:25	Santa Cruz	sc-292494	Granulosa cell (atretic follicle)
VEGF (vascular endothelial growth factor)	Rabbit	1:50	Abcam	ab46154	Corpus luteum
Secondary antibodies					
Alexa Flour 488	Donkey anti goat	1:50	Life Technologies	A11055	
Alexa Flour 488	Goat anti rabbit	1:50	Life Technologies	A11008	
Alexa Flour 488	Goat anti chicken	1:50	Life Technologies	A11039	
Alexa Flour 594	Goat anti rabbit	1:50	Life Technologies	A11012	
Alexa Flour 594	Donkey anti goat	1:50	Life Technologies	A11058	
Alexa Flour 594	Goat anti chicken	1:50	Life Technologies	A11042	
